# Comparative transcriptome analysis of stylar canal cells identifies novel candidate genes implicated in the self-incompatibility response of *Citrus clementina*

**DOI:** 10.1186/1471-2229-12-20

**Published:** 2012-02-14

**Authors:** Marco Caruso, Paz Merelo, Gaetano Distefano, Stefano La Malfa, Angela Roberta Lo Piero, Francisco R Tadeo, Manuel Talon, Alessandra Gentile

**Affiliations:** 1 Dipartimento di Scienze delle Produzioni Agrarie e Alimentari, Università degli Studi di Catania, Via Valdisavoia 5, 95123 Catania, Italy; 2 Institut Valencià d'Investigacions Agràries - Centre de Genómica, Carretera Montcada de l'Horta-Náquera Km. 4,5, 46113 Montcada de l'Horta (València), Spain

## Abstract

**Background:**

Reproductive biology in citrus is still poorly understood. Although in recent years several efforts have been made to study pollen-pistil interaction and self-incompatibility, little information is available about the molecular mechanisms regulating these processes. Here we report the identification of candidate genes involved in pollen-pistil interaction and self-incompatibility in clementine (*Citrus clementina *Hort. ex Tan.). These genes have been identified comparing the transcriptomes of laser-microdissected stylar canal cells (SCC) isolated from two genotypes differing for self-incompatibility response ('Comune', a self-incompatible cultivar and 'Monreal', a self- compatible mutation of 'Comune').

**Results:**

The transcriptome profiling of SCC indicated that the differential regulation of few specific, mostly uncharacterized transcripts is associated with the breakdown of self-incompatibility in 'Monreal'. Among them, a novel F-box gene showed a drastic up-regulation both in laser microdissected stylar canal cells and in self-pollinated whole styles with stigmas of 'Comune' in concomitance with the arrest of pollen tube growth. Moreover, we identify a non-characterized gene family as closely associated to the self-incompatibility genetic program activated in 'Comune'. Three different aspartic-acid rich (Asp-rich) protein genes, located in tandem in the clementine genome, were over-represented in the transcriptome of 'Comune'. These genes are tightly linked to a DELLA gene, previously found to be up-regulated in the self-incompatible genotype during pollen-pistil interaction.

**Conclusion:**

The highly specific transcriptome survey of the stylar canal cells identified novel genes which have not been previously associated with self-pollen rejection in citrus and in other plant species. Bioinformatic and transcriptional analyses suggested that the mutation leading to self-compatibility in 'Monreal' affected the expression of non-homologous genes located in a restricted genome region. Also, we hypothesize that the Asp-rich protein genes may act as Ca^2+ ^"entrapping" proteins, potentially regulating Ca^2+ ^homeostasis during self-pollen recognition.

## Background

Among the citrus species, several pummelos (*Citrus grandis *L. Osbeck) and mandarin-like varieties are self-incompatible [1]. Clementine mandarin (*Citrus clementina *Hort. ex Tan.), derived from an uncontrolled cross between a sweet orange and a mandarin, is probably the most widespread citrus species showing self-incompatibility (SI). It is characterized by gametophytic SI, with the pollen tubes stopping their growth in the upper or middle style [2,3]. Moreover, in this species SI is coupled with a variable degree of parthenocarpy. SI and parthenocarpy in citrus are very important traits for fruit production because they result in seedless fruits, which have a higher value in the markets compared to the seeded ones. Therefore, understanding the molecular basis of SI would be important to plan marker-assisted breeding to obtain new seedless genotypes.

Despite the importance of this trait, the genetic basis are still poorly understood and the key genes of SI have not been identified yet. The study of populations segregating for SI might be definitely a powerful strategy to give new insights into its genetic basis. However, the difficulties to obtain and characterize appropriate populations, with a variable degree of parthenocarpy and female/male sterility observed in the progeny might limit this approach. Such kind of strategy for the identification of the S-locus was carried out analyzing several crosses among different citrus cultivar and accessions with Got-3 isozyme, which is thought to be linked with the S-locus [4], providing only a rough estimation of their possible S-genotype.

In recent times, different research groups attempted to better understand SI and pollen-pistil interaction in several citrus genotypes, mainly trying to characterize putative homologs of key genes and proteins of already characterized SI systems. Gentile and colleagues [5] reported the involvement of Ca^2+^-dependent transglutaminase (TGase) in the self-incompatible response in pummelo, as already reported for *Rosaceae *[6]. Regarding the S-locus genes, a S-like RNase has been isolated from 'Zigui shatian' pummelo [7], however the authors suggested that this gene might play an important role during ovary senescence rather than in the incompatibility response. Another S-like RNase has been isolated from a mandarin variety and was partially characterized [8], but it's not still clear whether this gene is the key determinant for the self-incompatible response.

To overcome these limits, the transcriptome analysis of natural mutants displaying contrasting compatibility behaviour might be more effective to better understanding the molecular basis controlling the progamic phase in citrus. Over the last decade, the genome and/or transcriptome analysis of natural or induced citrus mutants have been a powerful strategy to study the molecular basis of agronomically important traits, such as ripening period, fruit pigmentation, seedlessness and other traits related to quality [9-12]. Concerning SI, a few citrus natural mutants displaying different sexual behaviour with respect to their original varieties have been identified and characterized [13-16]. Differences between the mutants and the original cultivars were related to differences in pollen [13], style [15] or ovary [14] functionality. In some cases, different behaviours during the progamic phase were associated to abnormal embryo development [13,16]. Therefore, it seems clear the mechanisms preventing fertilization are different in these genotypes, so it's reasonable to hypothesize that the mutations affected different genes or pathways implicated in reproduction.

Recently we chose two clementine clones with contrasting behaviour relating to self-pollen recognition ('Comune', self-incompatible; and 'Monreal', self-compatible mutation of 'Comune' [17]) as a model to identify candidate genes implicated in pollen-pistil interaction. Histological assays and analysis of pollen tube kinetics were performed to study the pollen tube behaviour in the two genotypes, and to assess whether the breakdown of SI in 'Monreal' was caused by changes in pistil or pollen functionality. The analysis demonstrated that the 'Monreal' mutation affected pistil functions, since pollen tubes of the two varieties grew equally in the pistils of self-compatible mutant, while 'Comune' rejected the pollen of both varieties, recognizing the pollen of 'Monreal' as self-pollen [15]. A first transcriptome comparison was conducted analyzing whole styles with stigmas, leading to the identification of a first set of genes differentially expressed in non-pollinated flowers and during pollen-tube elongation in self-pollination condition [15] including stress related genes, and transcripts related to Ca^2+ ^and hormone signalling. Surprisingly, a relatively high number of gene tags of different classes of retrotransposons were isolated, indicating their possible activation in response to pollination. However, the cDNA-AFLP analysis covered only part of the transcriptome and, due to the presence of pollen tubes growing along the pistil, the isolated gene tags were presumably not pistil-specific.

Here we describe a complementary approach to identify another set of candidate genes in 'Comune' and 'Monreal', to provide a better view of the molecular aspects related to citrus reproduction. We used laser capture microdissection (LCM), which has been efficiently used to investigate several aspects of plant reproductive biology [18-21]. In our approach, LCM was coupled to microarray analysis to identify genes specifically expressed in the stylar canal cells (SCC). We demonstrate that our transcriptomic survey of SCC is an efficient strategy to discover candidate genes involved in the SI response in clementine.

## Results

### Laser microdissection of SCC and microarray analysis

**Figure 1:**
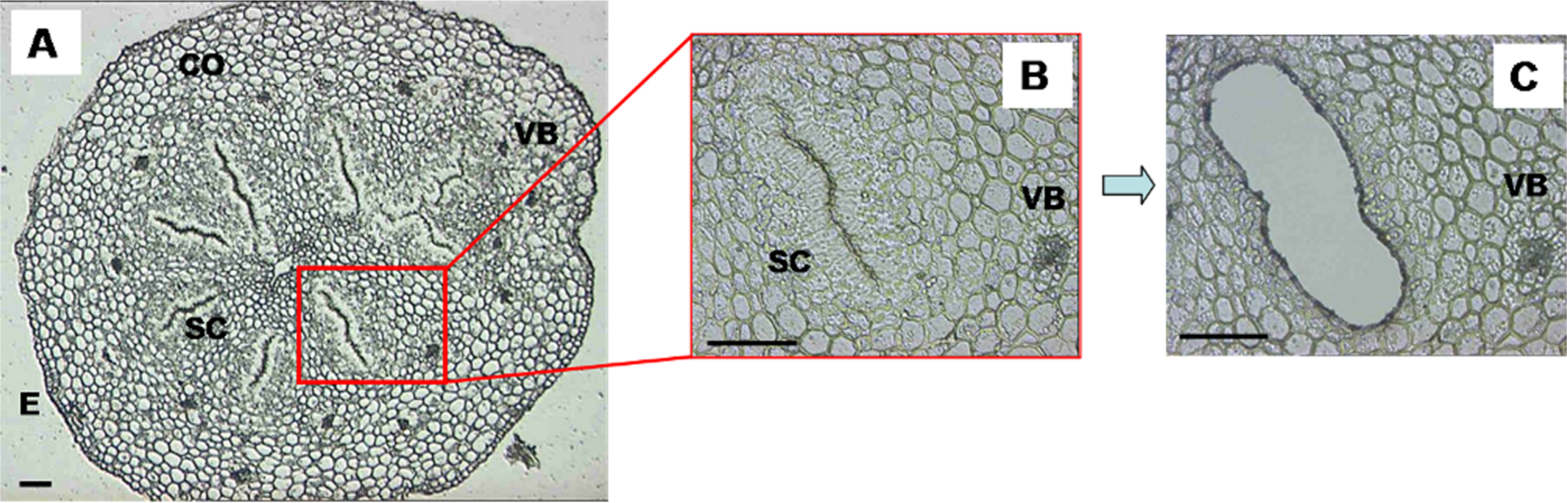
**Isolation of stylar canal cells using Laser capture microdissection (LCM)**. 10 μm-thick transversal section of 'Comune' clementine style (A), before (B) and after (C) laser microdissection. CO: cortex; E: epidermis; SC: stylar canals; VB: vascular bundles. Scale bar 100 μm.

Clementine pistil is composed of 10 or 11 fused carpels, which are assembled around an inner channel, and all sharing a common wet stigma that bears unicellular and multicellular papillae. Stylar canals are layered radially and lead to the ovary locules. Each canal is bordered by rectangular papillar cells, described in lemon [22] and 'Nova' mandarin [23]. SCC are placed with their long axis orthogonal to the canal. Although in some cases it is possible to see few pollen tubes growing within the inner channel [23] the majority of pollen tubes grows inside the stylar canals. As a result, these cells play a pivotal role in pollen-pistil interaction. To isolate the SCC, 10 μm transversal sections were cut from OCT (optimal cutting temperature)-embedded styles with stigmas in the upper third of the style before pollen tubes arrive. Once we checked the integrity of the embedded tissues, we performed the LCM of the SCC (Figure 1), discarding other tissues such as parenchyma and vascular bundles. Also, sections from the stigma were discarded, since stigma is not the site where SI reaction occurs [15]. Around 10,000 cells were microdissected from each biological replicate. The isolated RNA was not sufficient for the microarray hybridizations, therefore a two-round RNA amplification was performed. Additional file 1 shows the Agilent Bioanalyzer profiles of the biotin-labeled amplified RNA (aRNA) after the second round of amplification. aRNAs ranged from 200 to 2000 bp, with a peak around 600 bp, indicating the good quality of the aRNA after the in vitro transcription protocol.

Biotin-labeled aRNA was hybridized on the Affymetrix Citrus GeneChip^® ^which contains 30,171 probe sets representing up to 33,879 citrus transcripts from several citrus species and hybrids. The array is estimated to represent about 15,500 genes [24].

The three biological replicates of 'Comune' and 'Monreal' were compared using the Rank Products (RP) method. RP is a non-parametric analysis method which has been proven to be particularly sensitive in case of small number of biological replicates and large between-study variation [25]. The last aspect is of outstanding importance in our experiment, because sample processing might increase variability among replicates [26]. RP analysis detects genes that are consistently found among the most strongly up-regulated or down-regulated genes in a number of replicate experiments [27]. In our experiment, the comparison of transcriptomes of highly specific cells at the same physiological conditions resulted in a low number of differentially expressed genes identified between the self-incompatible and the self-compatible genotypes (Table 1). Specifically, 10 genes were over-represented in the SCC of the self-incompatible 'Comune', while 7 were over-represented in 'Monreal'. In general the differentially expressed genes showed a significant fold change-difference, especially in the case of the transcripts preferentially expressed in 'Comune'. They did not reveal any functional enrichment and were not homologous to genes previously characterized as involved in pollen-pistil interaction. Actually, many of the candidate genes were not previously annotated in other plant species.

**Table 1:** List of genes showing differential expression between 'Comune' (self-incompatible) and 'Monreal' (self-compatible) stylar canal cells

Probe set	Putative function	Fold change	PFP	*P *value
**Over-represented in the self incompatible (Comune)**				
Cit.7568.1.S1_at	putative F-box	37.14	0	0
Cit.11563.1.S1_at	No homology with functionally annotated proteins	16.15	0	0
Cit.5456.1.S1_at	No homology with functionally annotated proteins	4.72	0.06	0
Cit.5776.1.S1_s_at	No homology with functionally annotated proteins	11.99	0.002	0
Cit.7855.1.S1_at	No homology with functionally annotated proteins	14.02	0	0
Cit.4399.1.S1_s_at	Oligopeptide transporter-ISP4-like protein	17.05	0	0
Cit.24884.1.S1_at	Thioredoxin (TRX)-like [2Fe-2S] Ferredoxin (Fd) family	4.98	0.04	0
Cit.7174.1.S1_at	Aspartyl protease	5.39	0.03	0
Cit.19302.1.S1_at	Chalcone synthase	5.4	0.04	0
Cit.18732.1.S1_s_at	PSBS; photosystem II 22 kDa protein	5.71	0.06	0
**Over-represented in the self compatible (Monreal)**				
Cit.12037.1.S1_at	No homology with functionally annotated proteins	5.74	0.01	0
Cit.18491.1.S1_at	40S Ribosomal protein S24	3.94	0.02	0
Cit.7192.1.S1_at	Glycosyltransferase, CAZy family	4.34	0.03	0
Cit.1968.1.S1_s_at	Terpene synthase	4.71	0.04	0
Cit.9890.1.S1_s_at	GASA gibberellin regulated cysteine-rich protein	4.8	0.02	0
Cit.29299.1.S1_at	similar to uncharacterized Arabidopsis mitochondrial gene ATMG00030	4.35	0.04	0
Cit.8702.1.S1_s_at	Extensin	3.85	0.05	0

PFP, percentage of false-positives

**Figure 2:**
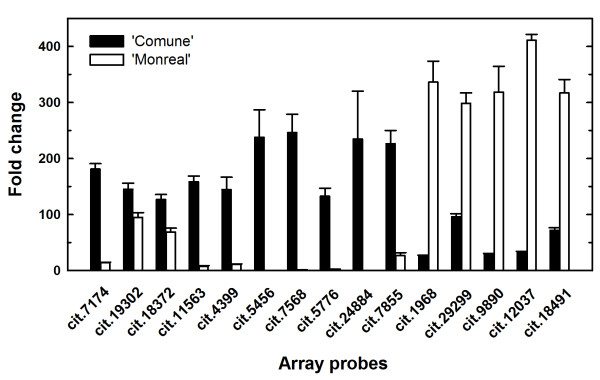
**Validation of the differentially expressed genes**. Relative expression levels of 15 unigenes in 'Comune' and 'Monreal' stylar canal cells determined using qRT-PCR, validating the microarray results. All genes are listed by their probe set ID of the Affymetrix Citrus GeneChip. Error bars indicate standard deviations from the mean.

For Real time quantitative RT-PCR (qRT-PCR) validations of the microarray results (Figure 2), total RNA was isolated from separate bulks of SCC, from which two-round unlabeled aRNA was synthesized. All the 'Comune' up-regulated genes were validated by qRT-PCR. Regarding the 'Monreal' over-represented genes, only one was not validated (cit.8702, corresponding to an extensin, data not shown), while cit.7192 was discarded for the presence of unspecific PCR products.

### An F-box is the most up-regulated gene in the SCC of the self-incompatible genotype

cit.7568 was the unigene which showed the highest fold-change value, with more than 37-fold over-representation in 'Comune' (Table 1). This unigene is represented in the GeneChip by a single clone of a *C. sinensis *callus library. Attempts to get a deduced amino acid sequence of the cit.7568 unigene led to no reliable predictions. Actually, partial cDNA amplification and sequencing of cit.7568 revealed that the transcript expressed in the SCC of 'Comune' was 209 bp longer, sharing higher similarity to a single EST from *C. sinensis *early-developing fruits [GenBank: EY696233]. Sequencing of the genomic region surrounding the Open Reading Frame (ORF) revealed allelic variation, and translation evidenced a non-functional allele, having 100% homology with the cit.7568 locus of the clementine haploid genome, and a functional allele, which encodes an intronless 268 amino acids F-box protein [GenBank: JN885720]. A 1.5 kb region spanning the putative F-box locus was sequenced but no polymorphisms were detected between 'Comune' and 'Monreal', indicating that the differential transcriptional regulation was not related to nucleotide polymorphisms. BlastX search showed that the predicted functional ORF shared homology to a poplar putative F-box protein [Uniprot: B9H9G5]. The putative Arabidospis ortholog (At5g04010) was annotated as a non-specific F-box. Interproscan identified a single F-box domain Skp2-like (IPR022364) and no additional protein motif structures. The F-box domain was similar to the one of SLEEPY (SLY1) F-box of Arabidopsis (At4g24210), that interacts with DELLA proteins and is involved in GA signalling [28]. However, no evident homology with SLY1 was observed outside the F-box domain.

### Three aspartic acid-rich (Asp-rich) protein encoding genes are preferentially expressed in self-incompatible condition

**Figure 3:**
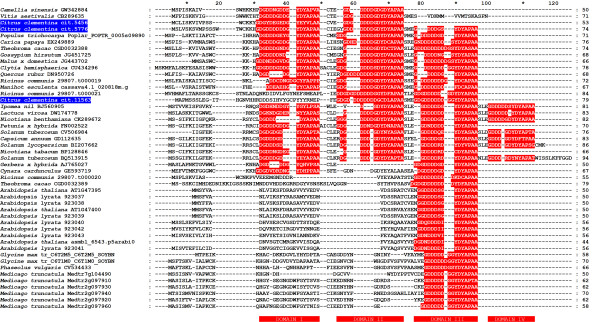
**Alignment of the Asp-rich protein genes**. Comparison of the amino acid sequences of the Aspartic-acid rich (Asp-rich) proteins from *Citrus clementina *(cit.5456, cit.5776, and cit.11563) with amino acid sequences deduced from putative genes and ESTs expressed in various plant species and in the jelly fish *Clytia hemisphaerica*. The Asp-rich domains (I to IV) in all plant and animal Asp-rich proteins are highlighted. Peptides were aligned with CLUSTALX [29] and the results displayed with GENEDOC.

Bioinformatic analyses added remarkable information regarding three unigenes found over-represented in 'Comune' (cit.11563, cit.5456 and cit.5776) and showing no functional annotation in available databases. The isolated genes represent three complete ORFs encoding small proteins containing 79, 53 and 74 amino acids, respectively. The deduced protein sequences share high homology degree and are characterized by an extremely high percentage of aspartic acid residues ranging from 19% (cit.5456) to 27% (cit.11563 and cit.5776) providing very low values of pI (3.64, 3.54 and 3.66, respectively). The alignment of the deduced amino acid sequences with other homologous proteins isolated from different plant sources shows that no significantly conserved sequences are detected in the first half of the N-terminal region, whereas two highly conserved motifs are detected both in the second half of the N-terminus and in the C-terminus of the proteins. The first conserved motif is a poly-aspartate assembly usually spaced out by glycine residues followed by a second YDYAPAA motif, both of them being still uncharacterized. The aforesaid motifs can be repeated in tandem along the protein sequence as observed in cit.5776 that exhibits three poly-D/G + YDYAPAA motifs along the sequence (Figure 3). Interproscan search did identify neither known functional domains nor signal peptides. Moreover, secondary structure prediction studies carried out upon the three deduced protein sequences revealed that, except small N-terminus regions likely folded as β-sheet, they are randomly coiled leaving the aspartate residues exposed in the surrounding environment (data not shown).

Another interesting characteristic was identified analyzing the location of the Asp-rich protein genes in the recently released draft of the clementine (v0.9) and orange (v.1) genomes (Haploid Clementine Genome, International Citrus Genome Consortium, 2011, http://www.phytozome.net/clementine; Sweet Orange Genome Project 2010, http://www.phytozome.net/citrus). That is, the 3 Asp-rich protein genes are located in tandem in a region of about 11 kb. Specifically, the Asp-rich protein genes are positioned in scaffold 9 of the clementine genome (Table 2), and in scaffold 5 of the sweet orange genome. In both genomes this region is highly conserved, with minor differences located in the uncoding regions. The main one is represented by a deletion of 616 bp in clementine, confirmed by PCR amplification and sequencing, in the region between cit.11563 and cit.5456 (data not shown). The Asp-rich protein genes are supported by a relatively low number of ESTs, for the most part found in non-flower-specific tissues subjected to stresses (Table 2).

**Table 2:** Putative aspartic acid-rich (Asp-rich) protein genes found in different species, with information on the number of associated ESTs supporting their expression in different tissues, and total number of ESTs for each species

Gene ID	Species	Db	Genome location	**EST no**.	Tissue	**EST total no**.
cit.11563	*C. clementina*	H, P	scaffold_9: 2625370-2627369	26	Callus, seedling, leaf (healthy and subjected to biotic and abiotic stress), mixed tissues	558387
cit.5456	*C. clementina*	H, P	scaffold_9: 2630019-2630586	13	Root challenged with nematode or abiotic stresses, seedling, callus, ovaries (abscission zone), cambium	558387
cit.5776	*C. clementina*	H, P	scaffold_9: 2635345-2635854	6	Roots with iron deficiency, seedling, CTV infected leaves	558387
29807.t000019	*R. communis*	P	29807: 160071-160289	0		62592
29807.t000020	*R. communis*	P	29807: 162893-163060	0		62592
29807.t000021	*R. communis*	P	29807: 166312-166566	0		62592
POPTR_0005s09890	*P. trichocarpa*	P	scaffold_5: 6981801-6981953	1	Bark challenged with insects	163281
Medtr2g097910	*M. truncatula*	P	MtChr2: 23065990-23066574	0		281422
Medtr2g097920	*M. truncatula*	P	MtChr2: 23068445-23068814	1	Virus-infected leaves	281422
Medtr2g097930	*M. truncatula*	P	MtChr2: 23070933-23071212	0		281422
Medtr2g097940	*M. truncatula*	P	MtChr2: 23073825-23074037	0		281422
Medtr2g097960	*M. truncatula*	P	MtChr2: 23078947-23079251	1	Flowers, early seeds, late seeds and stems	281422
Medtr7g104220	*M. truncatula*	P	MtChr7: 23576194-23576346	0		
Medtr7g104490	*M. truncatula*	P	MtChr7: 23711526-23711678	0		281422
AT1G47395	*A. thaliana*	P	Chr1: 17383034-17383408	2	Mixed library of flower, leaf and root	1529700
AT1G47400	*A. thaliana*	P	Chr1: 17385717-17386186	1	Inflorescence	1529700
asmbl_6543.p5arabi0	*A. thaliana*	P	Chr1:17389096-17389541	37	Mixed	1529700
923037	*A. lyrata*	P	scaffold_1: 23646793-23646985	0		583
923038	*A. lyrata*	P	scaffold_1: 23656301-23656493	0		583
923039	*A. lyrata*	P	scaffold_1: 23659179-23659371	0		583
923040	*A. lyrata*	P	scaffold_1: 23667427-23667637	0		583
923041	*A. lyrata*	P	scaffold_1: 23669301-23669511	0		583
923042	*A. lyrata*	P	scaffold_1: 23670810-23671020	0		583
923043	*A. lyrata*	P	scaffold_1: 23672121-23672332	0		583
C6T1M0	*G. max*	U	Gm05: 8142747-8142956	14	Mixed, root, cotyledon, leaf, seedlings treated with salicylic acid, whole plant	1461624
C6T2M5	*G. max*	U	Gm06: 21775640-21775813	7	Mixed, mixed stressed tissues, root, pod, flower, tissue culture	1461624
CGD0032389	*T. cacao*	CGD	super_9: 9549794-9550390	12	Pod, wood, leaf, flower, cushion	159996
CGD0032388_alt	*T. cacao*	CGD	super_9: 9552787-9553408	2	Leaves sprayed with the defense elicitors	159996
cassava4.1_020818m.g	*M. esculenta*	P	scaffold07478: 525348-525861	6	Mixed tissues from water stressed plant	80631
DW225255	*G. hirsutum*	G		3	Stem, mixed (meristematic region, very young fiber, roots, stem)	274247
JG443702	*M. × domestica*	G		10	Young leaves inoculated with *Marssonina coronaria*, flower, fruit, bud, leaf	324742
GD112635	*C. annuum*	G		4	Callus	118060
BQ513915	*S. tuberosum*	G		3	Mixed	249616
CV506984	*S. tuberosum*	G		1	Mixed floral	249616
BP128846	*N. tabacum*	G		1	Cell culture	332667
CK289672	*N. benthamiana*	G		1	Abiotic and biotic stress-treated leaves, callus tissue and root tissue	56080
BI207662	*S. lycopersicum*	G		2	Suspension cultures	299460
BJ560905	*I. nil*	G		3	Flowers and flower buds	62282
GW342884	*C. sinensis*	G		3	Leaves induced by *Ectropis obliqua *feeding	20,277
CV534433	*P. vulgaris*	G		1	Nodules	116,836
AJ765027	*G. hybrida*	G		1	Flower organ (pappus bristles)	16997
DN950726	*Q. robur*	G		1	Tissue culture growing 2 days in hypertonic medium	81671
EX249889	*C. papaya*	G		1	Flowers after meiosis	77393
CU434296	*C. hemisphaerica*	G		1	Not specified	85991
CB289635	*V. aestivalis*	G		3	Leaves	2101
DW174778	*L. virosa*	G		1	Mixed	30068
FN002522	*P. × hybrida*	G		2	Roots, developing ovaries	50705
GE593719	*C. cardunculus*	G		1	Green globe	36323

*EST no*. refers to ESTs found in dbEST database which typically showed query coverage and percentage of identity above 90%. *EST total no*. refers to the total number of ESTs deposited in the dbEST database of NCBI until August 2011. *Db *refers to the database from which the information regarding the Asp-rich protein genes were retrieved. H, Harvest database; P, Phytozome; G, GenBank; U, Uniprot; CGD, Cacao Genome Database

Carrying out similarity searches among the sequenced plant genomes we identified Asp-rich homologs in *Arabidopsis thaliana, Arabidopsis lyrata, Populus trichocarpa, Medicago truncatula, Glycine max, Ricinus communis, Theobroma cacao *and *Manihot esculenta*. As in citrus, the Asp-rich protein genes are usually in tandem, with the number of linked genes depending on each species and varying from 2 to 7. Exceptions are represented by poplar and cassava, where single copies are present. Moreover, soybean contains two putative Asp-rich proteins which are placed in different chromosomes, while in *Medicago*, in addition to 5 clustered Asp-rich located in chromosome 2, there are two unclustered identical Asp-rich in chromosome 7 (Medtr7g104220 and Medtr7g104490) which are elements of a duplicated region. *M. truncatula *and *A. lyrata *possess the highest number of Asp-rich paralogs (seven and six respectively), while *A. thaliana *displays three clustered Asp-rich proteins; two are predicted in the TAIR genome browser, release 9 (At1g47395 and At1g47400), while the third one has no Gene ID but was predicted using Fgenesh and is supported by 37 ESTs. In some cases, as in *A. thaliana*, gene predictions are supported by ESTs, while in others, such as *Ricinus *and *Medicago*, there are no EST corresponding to the prediction. The low EST support found in this species is probably due to the relatively low number of ESTs deposited in the databases, but also imply the highly specific role of this genes in specific metabolic processes.

Another search was performed against Uniprot and dbEST databases to identify other Asp-rich homologs and gene tags from additional species (Table 2). In the Asp-rich homologs, aspartate is still the prevalent residue, with an average percentage of 22% in the amino acids composition of the predicted proteins. Also, as the citrus Asp-rich proteins they are characterized by a short length (between 44 and 94 residues), by a variable number of poly-D/G domains (from one to four), and the majority presents the polyD/G + YDYAPAA at the C-terminus (Figure 3). As in the case of citrus, most of the identified ESTs were generated from stressed tissues, and in some cases from flowers, pointing out to a possible conserved role in different species. Interestingly, one EST belongs to a cDNA library from the jelly fish *Clytia hemisphaerica *suggesting that the role of this genes might not be plant-specific.

**Figure 4:**
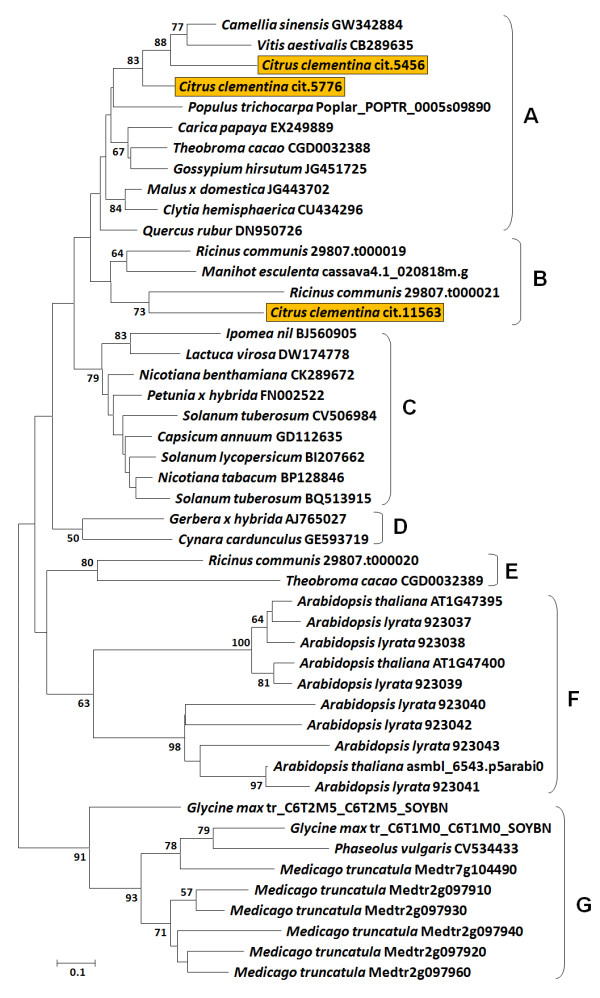
**Phylogenetic analysis of the Asp-rich protein genes**. The Neighbor joining tree based on p-distance and pairwise deletions of gaps shows phylogenetic relationship among the putative Asp-rich proteins identified in different species. The numbers at the branch points indicate bootstrap support values above 50% (1,000 replicates). Based on Neighbor joining clustering, Asp-rich proteins were tentatively grouped into seven main families, from A to G. The citrus Asp-rich proteins are highlighted.

The phylogenetic analysis was conducted aligning the whole predicted protein sequences using ClustalX (Figure 3). The neighbor joining (NJ) tree separated the Asp-rich proteins into seven major groups, which are tentatively called subfamilies from A to G (Figure 4). Proteins with equal number of poly-D domains or similar domain organization usually grouped together. The A and B subfamily are divided into subclades comprising Asp-rich proteins which possess two or three conserved domains scattered along their sequence and a high percentage of Asp residues (from 19% to 32%). The C family consists of Asp-rich proteins from Solanaceae, plus a subcluster including *Lactuca *and *Ipomoea *proteins. Most of the proteins of the C group possess 4 Asp-rich domains. D and E are small subfamilies which comprise, respectively, Asp-rich proteins from *Gerbera x hybrida *and *Cynara cardunculus *(D), and from castor bean and cacao (E). *Arabidospis *proteins, containing a single poly-D domain at the C-terminal region, are all grouped in the subfamily F, while the Asp-rich proteins from legumes clustered in the subfamily G. The predicted sequences belonging to the groups F and G have a relatively low percentage of Asp (from 10% to 20%). The paralogs of *M. truncatula *and of the two Arabidopsis species display a high level of similarity and are tightly grouped. On the contrary, higher variability in length and number of the Asp-rich domains was observed in citrus, cacao and castor bean. It is noteworthy that the paralogs of these species belong to different sub-clades, suggesting a possible sub-functionalization.

### The genomic region surrounding the Asp-rich protein genes is conserved among genomes

Further structural analysis of the genomic regions surrounding the Asp-rich protein genes evidenced another remarkable feature. Specifically, we identified a DELLA protein gene about 40 kb downstream the Asp-rich protein genes. DELLA was found to be differentially expressed in a previous transcriptome profiling [15]. In particular, its mRNA levels were higher in 'Comune' pollinated styles with stigmas compared to 'Monreal' ones.

**Figure 5:**
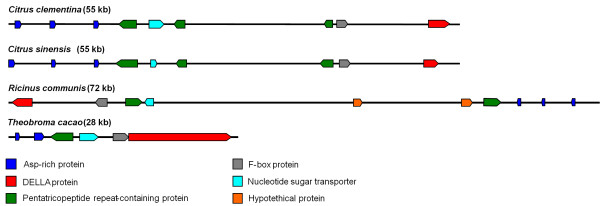
**Collinearity of a 55 kb genomic region of clementine and sweet orange with homologous regions of castor bean and cacao genomes**. The region contains three Asp-rich protein genes and a DELLA gene which are up-regulated during self-pollen rejection in 'Comune' clementine.

The region between the Asp-rich protein genes and DELLA is highly conserved in *C. clementina *and *C. sinensis *genomes. Moreover, this segment showed collinearity with the genomes of two unrelated plants such as cacao (*T. cacao*) and castor bean (*R. communis*) (Figure 5). In the four genomes, the Asp-rich protein genes are in the same orientation of DELLA. Between the Asp-rich protein genes and DELLA, other predicted ORFs are present (Figure 5). The clusters of the four genomes share the main structure and gene orientation, with the presence of pentatricopeptide repeat (PPR) - containing proteins, an EamA-like nucleotide sugar transporter and an F-box gene located between the Asp-rich protein genes and DELLA. Not all the predicted genes are supported by expression data. The coding sequence of the F-box putative protein (represented by the grey box) upstream DELLA is not represented in the citrus GeneChip, and it has no significant similarity with the cit.7568 F-box.

### Histological and transcriptional changes during pollen-pistil interaction

**Figure 6:**
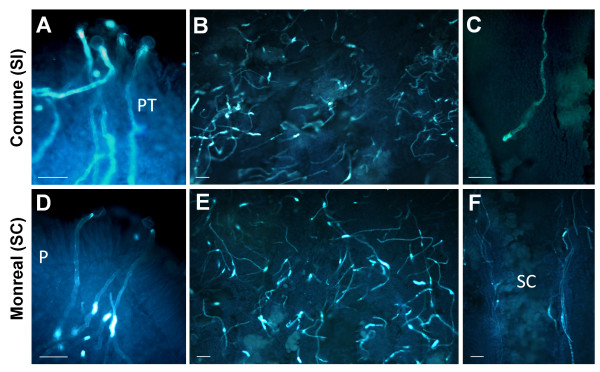
**Squash preparations of self-pollinated styles with stigmas of 'Comune' and 'Monreal'**. Similar behaviour of pollen germination (A, D; pictures taken 2 days after pollination) and pollen tube elongation (B, E; pictures taken 4 days after pollination) were observed in both genotypes during the early phases of pollen-pistil interaction. However, in 'Comune', pollen tubes stopped their growth in the middle style some five days after pollination (C), and no pollen tube was observed in the low style; in 'Monreal' they grew along the stylar canals (F, picture taken 6 days after pollination) and reached the base of the style 7 days after pollination. P: papillae; PT: pollen tubes; SC: stylar canals. All sections were stained by aniline blue. Scale bars: 50 μm.

**Figure 7:**
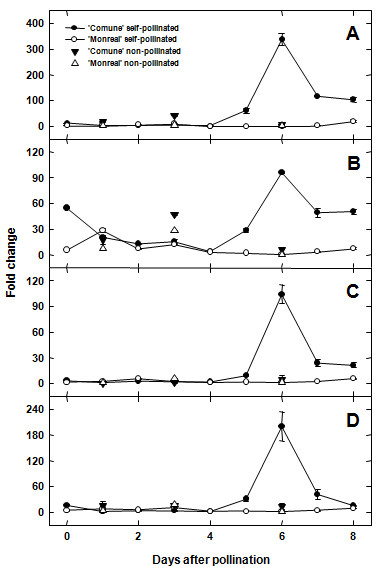
**Relative gene expression of selected genes during pollen pistil-interaction**. Genes are listed by their probe set ID of the Affymetrix Citrus GeneChip and by their tentatively assigned name. A: cit.7568 (F-box); B: cit.11563 (Asp-rich protein gene); C: cit.5456 (Asp-rich protein gene); D: cit.5776 (Asp-rich protein gene). Styles with stigmas were sampled from 0 (non-pollinated flowers) to 8 DAP. Control samples of non-pollinated flowers, collected at 1, 3 and 6 DAP, were added to the time course analysis to determine if the genes were induced by self-pollination. Error bars indicate standard deviations from the mean.

To investigate possible changes in mRNA levels during pollen germination and pollen tube elongation, a time course analysis was performed on whole styles with stigmas. We focused on the F-box-containing unigene cit.7568, which showed the highest fold change among the differentially expressed genes, and on the putative Asp-rich protein genes (cit.11563, cit.5456 and cit.5776). For this analysis, we collected non-pollinated flowers just prior to flowering (indicated as T0) as well as self-pollinated styles with stigmas from one to eight days after pollination (DAP), indicated as T1 to T8. Moreover, we investigated the expression patterns in non-pollinated flowers collected at T1, T3 and T6 to assess whether the genes were induced by pollination, or conversely, were developmentally regulated. A daily histological observation of self-pollinated styles with stigmas was performed on both varieties to monitor the rate of pollen tube elongation (Figure 6). This analysis was previously conducted on both genotypes [15]. However, since the pollen tube growth is highly influenced by environmental temperature, new microscopy observations were essential to detect possible coincidences between pollen tube behaviour of 'Comune' and 'Monreal' and transcriptional changes of the candidate genes. From T0 to T4, pistils of both varieties displayed similar performances regarding pollen tube germination and pollen tube elongation. Pollen germination was evident from T1 (Figure 6A-D), and until the 4^th ^DAP germinated tubes were at the level of the stigma (Figure 6B-E), some of them reaching the upper style. Between T0 and T4, a slight up-regulation of all the analyzed transcripts was observed in 'Comune', although mRNA levels of the 4 genes were generally low (Figure 7). The exception is represented by cit.11563, which was already some 9 fold up-regulated in 'Comune' at T0. After T4 we observed a marked difference both in pollen tube kinetics and gene expression. In 'Comune', the self-incompatible genotype, pollen tubes were still in the stigmatic canals, and only a few were recorded in the upper or middle style (Figure 6C). Conversely, in 'Monreal' a conspicuous number of pollen tubes grew along the middle style (Figure 6F), reaching the base of the style after 7 days. The differences in pollen tube behaviour coincided with differences in gene expression. From T5 a clear up-regulation of the 4 genes was observed in 'Comune', with a peak of expression at T6, while in 'Monreal' no changes were observed compared to the previous sampling dates (Figure 7). 'Comune' unpollinated flowers at T6 showed mRNA levels comparable to the ones of 'Monreal', indicating the self-incompatible condition elicited the up-regulation of the F-box and of the Asp-rich protein genes.

**Figure 8:**
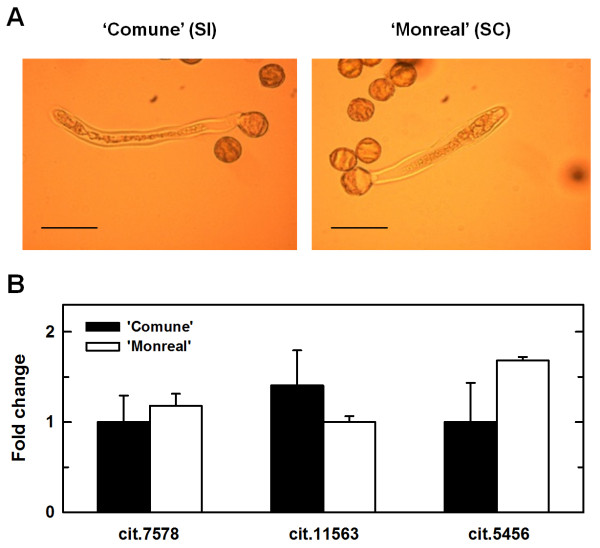
**qRT-PCR analysis of mRNA isolated from pollen tubes**. (A) Pollen tubes of 'Comune' and 'Monreal' grown in vitro after 24 h of culture. Scale bars: 50 μm. (B) mRNA levels of cit. 7568, cit.11563 and cit.5456 in pollen tubes grown in vitro. No expression was detected for cit.5776. Error bars indicate standard deviations from the mean. Expression levels were normalized against citrus Ubiquitin unigene contained in the GeneChip (AFFX-Cit-ubq11-3_x_at).

Additional qRT-PCR were performed on pollen tubes grown in vitro, to assess whether the different mRNA levels detected in the time course analysis might have been influenced by a differential expression at the level of pollen tubes. RNA was isolated from tubes grown for about 24 h, which showed similar rate of elongation between the two clementines (Figure 8A). Extremely low mRNA levels and no differential expression were observed in cit.7568, cit.11563 and cit.5456 (Figure 8B), while no expression was detected for cit.5776. The time course analysis was also performed to analyze the expression patterns of DELLA, which was preferentially expressed in 'Comune' styles with stigmas at T6 and T7 (Additional file 2).

## Discussion

In this study, LCM was used to spefically isolate SCC of two clementine genotypes differing for the SI response. 'Comune' is a widespread self-incompatible variety, while 'Monreal' is a self-compatible variety originated from a spontaneous bud mutation [17]. The microdissection of the SCC allowed to perform a highly specific study of the transcriptome of the cells implicated in the interaction between pistil and pollen tubes, with the main aim of identifying candidate genes involved in self-pollen rejection. The results of microarray analysis suggested that the differential regulation of few specific transcripts might have lead to the breakdown of SI in 'Monreal'. Based on functional information retrieved from the databases, these genes do not show any clear functional enrichment, and many of them have no annotation, making it difficult to compare our results with SI systems characterized on model plants. On the other hand, our experiment provided a new set of transcripts that are very likely to play a key role during the progamic phase in citrus. Searches in the databases revealed that most of these genes are not flower specific, suggesting that probably the mutation leading to the breakdown of SI did not affected the S-locus determinants. However, it is known that non S-locus genes are implicated in SI [30]. Most of the preferentially expressed genes were low-represented in EST databases and showed a weak expression in whole styles with stigmas before the SI reaction occurred. This indicates the usefulness of the LCM in the identification of highly specific and/or low expressed genes.

Flowers for LCM were sampled one day after pollination, when pollen was just germinating. Self-pollinations were performed to assess whether the presence of pollen in the stigma might have elicited a differential response of the SCC in self-compatible vs. self-incompatible conditions. So, while pollen tubes where still in the stigma, we targeted the rectangular SCC located in the upper/middle style, which is the site where incompatibility occurs in 'Comune', without contamination of pollen tubes. Although we did not analyze mRNAs of microdissected SCC of non-pollinated flowers, qRT-PCR demonstrated that slight differences in gene expression were also present in whole styles with stigmas before pollination, indicating that, at least in the cases of cit.7568, cit.11563, cit.5456 and cit.5776, the different mRNA levels in the SCC between 'Comune' and 'Monreal' were not induced by the pollen germination in the stigma.

Among the identified genes, our analysis focused on four unigenes over-represented in 'Comune' (cit.7568, cit.11563, cit.5456 and cit.5776). A time course analysis coupled to histological observation of pollen tube elongation was performed to correlate pollen tube behaviour with the difference in gene expression between 'Comune' and 'Monreal'. Until the 4th DAP we could not observe any clear differences in the mRNA level of the 4 analyzed genes, and no clear differences were evident in pollen tube behaviour between the two varieties. Form the 5^th ^DAP we observed an impressive up-regulation in the self-incompatible combination. Selected candidate genes showed a clear differential expression (even > 100 fold change) during pollen - style interaction (Figure 7), in agreement with histological observation (pollen tube growth in 'Monreal' and no growth in 'Comune'). The peak of up-regulation was evidenced in concomitance with the pollen tube arrest, suggesting that all the analyzed genes have a key role in the stop of pollen tube elongation. The up-regulation was clearly induced by pollen tubes in the styles, as confirmed with the comparison of the expression levels of non-pollinated and self-pollinated flowers at T6 (Figure 7). The candidate genes were weakly expressed in pollen tubes and no differences in the mRNA levels were observed between 'Comune' and 'Monreal' (Figure 8). As a result, it is unlikely that the transcriptome of pollen tubes influenced the mRNA levels detected in the time course analysis. In fact, our expression data demonstrate that the drastic changes in mRNA levels occurred in the stylar tissues as a response to self-incompatible pollen tubes. Also, the drastic up-regulation of the four genes should not represent a downstream response to SI, since these genes are already differentially regulated in SCC before SI occurs.

Bioinformatic analysis provided useful information to hypothesize the role of the four unigenes. cit.7568 probe set matched to a putative F-box protein gene sharing homology to an Arabidopsis F-box (At5g04010) annotated as non-specific [28]. This protein belongs to the C2 group of the F-box superfamily, of which SLY1 belongs. However, the similarity with SLY1 regards the F-box domain, while the C-terminus, responsible for targeting the substrate for ubiquitination, does not share any homology with SLY1.

F-box protein superfamily is one of the largest in plants [31,32]. These proteins are key regulators of proteolysis, conferring specificity to the SCF (Skp1, Cullin, F-box) E3 ubiquitin ligase complex, which is responsible for recognizing the substrate for ubiquitin-mediated protein degradation. Despite the fact that it is not possible to correlate the role of the novel clementine F-box gene with any characterized orthologs, database searches and transcriptional data revealed that the clementine F-box gene was not previously identified in clementine cDNA libraries and evidenced highly specific expression patterns, since it was almost 250 fold up-regulated in SCC (Figure 2) and about 50 to 300 fold up-regulated in whole styles with stigmas (Figure 7A) in concomitance with the arrest of pollen tube elongation. This drastic up-regulation, and the absence of identical ESTs in databases support the high specificity of this gene and point out to its possible involvement in highly specific proteolytic events occurring in the style during the self-incompatible response. Studies on the families where SI system have been already characterized (namely *Brassicaceae, Solanaceae *and *Papaveraceae*) showed that proteolysis has a key role during self-pollen rejection. Usually proteolytic events follow the initial SI signal perception leading to the eventual death of the male gametophyte [33]. Functional analysis will be necessary to shed light on the role of the clementine F-box during pollen-pistil interaction.

Another striking finding was the identification of the three putative Asp-rich protein encoding genes, cit.11563, cit.5456 and cit.5776, up regulated in 'Comune'. In addition to the high similarity in their primary structure, they co-localize in the clementine genome (Figure 5). The clustered genes displayed similar expression patters (Figure 7B, C, D) which might indicate that they are commonly regulated as already reported for other clusters [34,35]. Although their function has not been investigated, it is known that the expression of the *Arabidopsis *homologous At1g47400 is sharply up regulated in plants exposed to 3-(30,40-dihydroxyphenyl)-L-alanine (L-DOPA), a phytotoxic allelochemical [36]. In other studies, this gene turns out to be strongly induced by iron deficiency [37] and by treatment with the polycyclic aromatic hydrocarbon phenanthrene [38]. These results support the hypothesis that the genes encoding the Asp-rich proteins might be triggered in response to different types of stresses. Since other information is still lacking, we attempt to assign a putative function to the *Citrus *Asp-rich proteins and also propose a model which overall might explain the regulation of SI in the 'Comune' variety. Because of their richness of aspartic acid residues, the Asp-rich proteins are supposed to act as novel Ca^2+ ^"entrapping" proteins. Although they do not show sequence similarity with other well known Ca^2+ ^interacting proteins, such as calsequestrin, calreticulin and calmodulin, the Asp-rich proteins share with those proteins the aspartate residue abundance which has been related with the protein ability of Ca^2+^-binding [39]. Therefore, assuming that Ca^2+ ^levels play a decisive role during pollen-pistil interaction, as already reported for several plant species [40], the up-regulation of the Asp-rich encoding genes observed in 'Comune' might enhance the amount of proteins functioning as Ca^2+^-trap elements, and lead to an exceptional decrease in Ca^2+ ^availability, thus contributing to switch off the signal cascade usually induced by the increase in cytosolic Ca^2+ ^concentration or to the alteration of Ca^2+ ^gradient needed for pollen tube elongation. Pollen tube growth could represent, among others, the downstream physiological process dramatically affected by this missing triggering event. To support this hypothesis, it is worthwhile to mention that the aforesaid At1g47400 gene is up regulated in a T-DNA insertional mutant of a P-type ATPase cation pump, the *MALE GAMETOGENESIS IMPAIRED ANTHERS *(*MIA*); the mutant shows reduced male fertility and imbalanced cation homeostasis [41]. Moreover, a Ca^2+ ^pump (auto-inhibited Ca^2+^-ATPase - ACA), which presumably pumps Ca^2+ ^out the cytosol [42], is differentially regulated in the two genotypes during pollen-pistil interaction showing an up regulation in the 'Comune' genotype [15]. Further mandatory work will be undertaken to validate both the supposed role and the functioning model of pollen-pistil interaction in *Citrus *genotypes and, if they are proved, this might represent a case in which specific regulatory mechanism involving different loci rather than the *S*-locus could be co-responsible of the SI determination.

The integration of the transcriptomic data with the synteny analysis denoted a specific genome region containing a cluster of genes activated during self-pollen rejection (Figure 5). Specifically, the Asp-rich protein genes are linked to a DELLA gene which was previously isolated in the self-pollinated 'Comune' styles with stigmas [15] and showed a preferential expression in the self-incompatible genotype. This raise intriguing questions about the possibility that the non-homologous genes located in this genome region might contribute to a common function related to self-pollen rejection. Examples of co-expressed and functionally related gene clusters in eukaryotes have emerged over the last decade [34,43] and are likely to increase with the huge amount of data coming from the genome projects. The most investigated operon-like organizations in plants are secondary metabolic pathways [44], mostly implicated in plant defence response. Clustering appears to have occurred *de novo *through some form of convergent evolutionary process [43]. In our case, it is unlikely that the genes are clustered by chance, since this cluster is conserved in at least two other plant species, cacao and castor bean (Figure 5). The collinearity of the citrus genome segment comprising the Asp-rich protein genes and DELLA with other segments of unrelated genomes support the hypothesis that selection might have favoured the linkage of these genes. The advantage of clustering is related with the fact that tightly linked genes might be co-regulated at the levels of nuclear organization and/or chromatin [43,45]. The co-localization of the three up-regulated Asp-rich protein genes as well as DELLA in the scaffold 9 of the clementine genome v0.9 suggests that the mutation leading to self compatibility probably affected the functionality of tightly linked genes. Further transcriptional analyses will be carried out on the other genes surrounding the Asp-rich protein genes to study their possible role during different stages of the pollen-pistil interaction.

## Conclusion

LCM coupled to microarray analysis was definitely a powerful tool to identify candidate genes involved in self pollen rejection which have not been previously associated to SI. Data at the transcriptome level strongly suggested that a restricted number of differentially regulated transcripts are associated with self-pollen recognition. Although functional information is missing, we hypothesize that proteolysis and Ca^2+ ^homeostasis might be crucial for SI response in clementine, reflecting to some extent molecular events occurring in other SI systems. However, further work at the protein level will be necessary for understanding the role of the candidate genes during self-pollen recognition. Also, the unigenes represented in the GeneChip do not cover the whole transcriptome, consequently other strategies such as RNA-seq might help to improve the transcriptome coverage to identify additional genes implicated in self-pollen recognition.

## Methods

### Plant material

Plant material was collected from adult trees of 'Comune' clementine and its self-compatible natural mutation 'Monreal', grown at the experimental station of Consiglio per la Ricerca e la sperimentazione in Agricoltura (CRA) placed in Palazzelli, Lentini (SR), Italy.

### Laser microdissection of SCC

Styles with stigmas, collected 24 hours after self-pollination, were immediately snap-frozen in OCT embedding medium (Sakura Finetek, Zoeterwoude, Netherlands) in Peel-A-Way plastic embedding molds (Polysciences, Polysciences, Warrington, PA, USA). The embedded samples were stored at -80°C until used. Transversal sections 10 μm thick at the upper part of the style were cut with a Leica CM1900 cryostat (Leica Microsystems, Germany) at -20°C. Cryosections were mounted on PET-membrane-coated stainless steel slides (Leica Microsystems, Wetzlar, Germany) and processed as described previously [46]. Each slide contained 15-20 style sections. A Leica AS Laser Microdissection system (Leica Microsystems) was used for the isolation of stylar canals from transversal sections. Canals from the stigma were discarded to avoid contamination with pollen or pollen tubes. Microdissection was performed using the X40 magnification lens with the following settings: aperture: 6; intensity: 46; speed: 2; offset: 40; bridge: medium. SCC were collected in the cap of a 0.5 ml microtube filled with RLT buffer from the RNeasy Plus Micro Kit (Qiagen, Hilden, Germany).

### RNA isolation and amplification

Three biological replicates were prepared for each genotype. Each biological replicate consisted of bulks of about 200 microdissected areas (composed of an average of 50 cells) coming from two different molds. RNA isolation was performed from ~ 10,000 cells using the RNeasy Micro Kit (Qiagen) following the manufacturer's instructions. RNA samples were subjected to a two-round amplification TargetAmp™ 2-Round Biotin-aRNA Amplification Kit 3.0 (Epicentre Biotechnologies, Madison, WI, USA) following the manufacturer's instructions. Quality of the aRNAs was assayed by OD260/OD280 measurements, agarose gel electrophoresis and Agilent 2100 Bioanalyzer.

### Microarray analysis

Microarray experiments were performed using the GeneChip^® ^Citrus Genome Array (Affymetrix, Santa Clara, CA, USA). Ten micrograms of biotynilated aRNA were used for the chip hybridizations. Data obtained from the microarray experiment were processed using Robin [47], which consists in an easy to use graphical interface for microarray analysis functions from R/BioConductor. CEL files were normalized by Robust Multi-Array Analysis (RMA) method. All microarray data have been deposited in the Gene Expression Omnibus (GEO) database under accession GSE33014. The list of probe sets showing significant differential expression was calculated comparing the three replicates for each genotype using Rank Products method [25]. Percentage of false-positives (PFP) cut-off, corresponding to the false discovery rate, was set at 0.06 and calculated based on 100 permutations. Probe set information was retrieved from Harvest: Citrus database http://harvest.ucr.edu, http://www.harvest-web.org. Differentially expressed unigenes were subjected to Basic Local Alignment Search Tool (BLAST) search against the GenBank NR database. Interproscan http://www.ebi.ac.uk/Tools/InterProScan/ was used to identify conserved domains in the deduced protein sequences.

### Validation of differentially expressed genes

Two RNA isolations (one for each genotype) were performed from additional bulks of SCC for the validation of microarray results. Each RNA was extracted from 10,000 cells and amplified using TargetAmp™ 2-Round aRNA Amplification Kit 2.0 to generate unlabeled aRNA. Quality of the aRNAs was assayed by OD260/OD280 measurements and agarose gel electrophoresis. cDNA was synthesized from 500 ng aRNA using the Ready-to-go RT-PCR beads (GE Healthcare Technologies, Little Chalfont, UK) with primer dT. Before performing qRT-PCR, an RT-PCR was carried out to check the presence of possible unspecific products. The transcript levels were determined by real-time quantitative RT-PCR (qRT-PCR) using the ABI Prism 7000 Sequence Detection System (Applied Biosystems, Foster City, CA, USA) and the Power SYBR Green PCR Master Mix as previously described [15]. Real time was performed using three cDNA synthesis for each genotype, and each amplification was repeated twice. Primers used are listed in the Additional file 3. Primer pairs that did not reveal single dissociation peak were discarded. mRNA levels were calculated by standard-curve with a 5-fold dilution series and normalized against citrus Ubiquitin unigene contained in the GeneChip (AFFX-Cit-ubq11-3_x_at).

### Histological observations

Ten self-pollinated flowers for each genotype were collected every 24 hours from the first to the eighth day after pollination to monitor pollen germination and pollen tube kinetics. Squash preparations and microscopy observation were performed as described in Distefano et al. [15].

### Time course analysis

Selfed 'Comune' and 'Monreal' styles with stigmas were collected from 0 (virgin flowers) to 8 DAP. To check whether the expression patterns were influenced by pollen-pistil interaction, control samples of non-pollinated flowers were collected at 1, 3 and 6 DAP. Bulks of about 25 styles with stigmas were immediately stored in liquid nitrogen after collection and were used for total RNA isolation using RNeasy Plant Mini Kit according to the product manual (Qiagen). Total RNA was treated with RNase-Free DNase set (Qiagen) according to the manufacturer's instructions. Total RNA was amplified by PCR to confirm the absence of genomic DNA in the samples. cDNA synthesis was carried out starting from 2 μg total RNA. Real time runs and the data analysis were performed as described above. Two biological replicates were used for each sampling date, and all amplifications were repeated twice. Time course was carried out to evaluate expression patterns of 4 unigenes preferentially expressed in 'Comune' (probe set names cit.7568, corresponding to the SLY-like F-box gene; and cit.11563, cit. 5456, cit.5776, corresponding to the putative Asp-rich protein genes) as well as of a DELLA gene previously isolated by cDNA-AFLP from 'Comune' self-pollinated styles with stigmas [GenBank: GH733267].

### In vitro pollen tube germination and RNA isolation

About 30 flowers were collected just before anthesis and their petals and pistils were removed. The anthers were left to dehisce for 24 h at room temperature. The fresh pollen was cultured in Petri dishes in the liquid pollen germination and tube growth medium described by Mesejo et al. [48]. After 24 h of culture, pollen tubes were pelleted by centrifugation and harvested for RNA isolation using RNeasy Plant Mini Kit (Qiagen).

### Similarity searches and phylogenetic analysis of the Asp-rich protein genes

DNA sequences of citrus unigenes corresponding to the Asp-rich protein were blasted against the sequenced genomes available at the Phytozome website v 7.0 http://www.phytozome.net. Other sequences of Asp-rich homologs were retrieved from GenBank, Uniprot and the Cacao Genome Database http://www.cacaogenomedb.org.

ORF prediction was based on the genome browser data for each analyzed species as well as Fgenesh analysis http://www.softberry.com. All the predicted protein sequences (3 citrus Asp-rich plus 44 from other species) were aligned by ClustalX [29]. Alignments were analyzed using Mega 5 package [49] and a phylogenetic tree based on p-distance and pairwise deletions of gaps was constructed using the neighbor joining method with 1,000 bootstrap replicates.

### Identification of the cit.7568 F-box functional allele

A 1,448 bp fragment that span the cit.7568 locus was amplified in both genotypes using the primers provided in the Additional file 3. The region was divided in three PCR products, which were cloned into pGem-T Easy Vector (Promega, Madison, WI, USA) and sequenced, or directly sequenced, using an ABI310 genetic analyzer (Applied Biosystems). The sequence of the region spanning the cit.7568 locus was deposited in the NCBI GenBank database [GenBank: JN885720].

## Abbreviations

aRNA: Amplified RNA; Asp-rich: Aspartic acid-rich; DAP: Day(s) after pollination; EST: Expressed sequence tag; LCM: Laser capture microdissection; OCT: Optimal cutting temperature; ORF: Open reading frame; qRT-PCR: Real time quantitative RT-PCR; RP: Rank products; SCC: Stylar canal cells; SI: Self-incompatibility; SLY1: SLEEPY.

## Authors' contributions

MC performed LCM, RNA extractions and amplifications, microarray analysis, qRT-PCR validation of differentially expressed genes, time course analysis, microscopy observations, in vitro pollen germination assay, bioinformatics and phylogenetic analyses, and drafted the manuscript. PM optimized and assisted LCM and RNA amplification experiments. GD carried out samplings and tissue embedding for LCM analysis, assisted qRT-PCR analyses and microscopy observations. ARLP performed and supervised bioinformatic analyses, helped in the interpretation of the results and participated in the draft of the manuscript. SLM assisted experimental design and helped draft the manuscript. FRT conceived the overall research, designed and funded LCM and aRNA experiments, assisted microarray analysis and phylogenetic analysis of the Asp-rich protein encoding genes, and participated in the draft of the manuscript. MT and AG coordinated, supervised and funded the experiments, and critically revised the manuscript. All authors have read and approved the final manuscript.

## Supplementary Material

Additional file 1 **Agilent 2100 Bioanalyzer profiles of amplified RNAs (aRNAs)**. Profiles show the size distribution of aRNAs produced from the different biological replicates after two rounds of amplification. Top central, RNA size marker ladder (RNA 6000 Ladder, Applied Biosystems). On the right of each electropherogram is a gel image generated from aRNAs with a smear ranging from 200 to 2000 bp and a peak around 600 bp. Click here for file

Additional file 2 **Real time expression patterns of DELLA gene during pollen-pistil interaction in the self-compatible ('Monreal') and the self-incompatible ('Comune') genotypes**. Click here for file

Additional file 3 **List of primer pairs used for the real time quantitative RT-PCR analysis and for sequencing**. Click here for file
