# Supplementary figures and images for: Comparative transcriptome analysis of stylar canal cells identifies novel candidate genes implicated in the self-incompatibility response of Citrus clementina
Source: BMC Plant Biol. 2012 Feb 14;12:20. doi: 10.1186/1471-2229-12-20 (PMC3305554; doi:10.1186/1471-2229-12-20)

# Agilent 2100 Bioanalyzer – aRNA analysis

Ladder

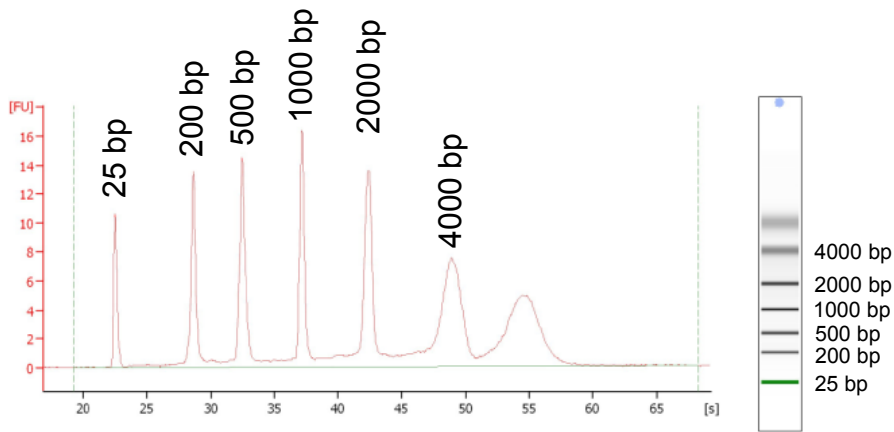

Comune 1

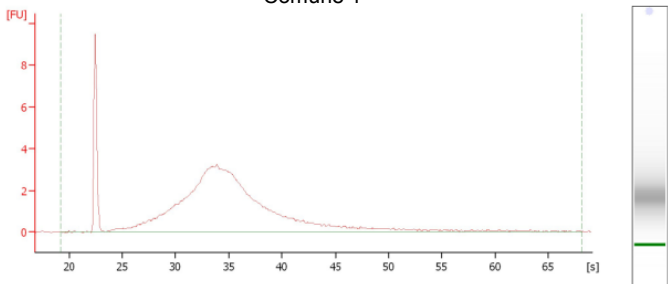

Monreal 1

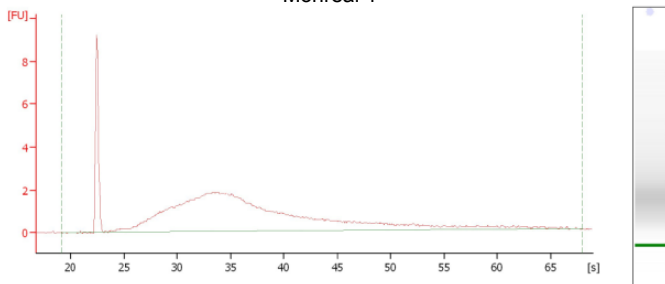

Comune 2

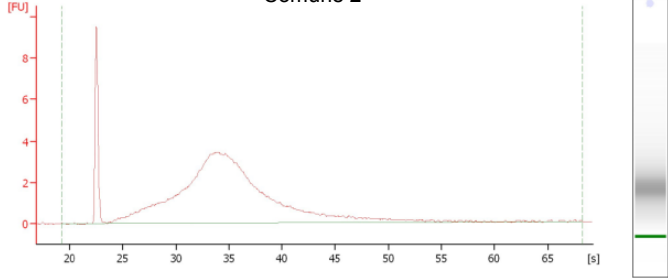

Monreal 2

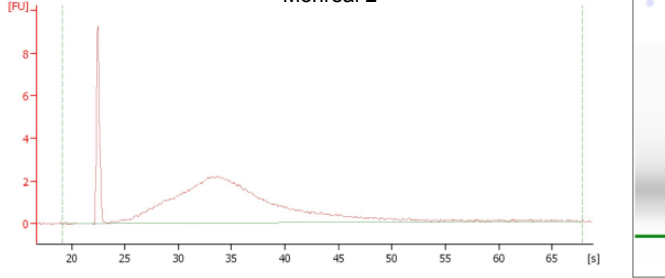

Comune 3

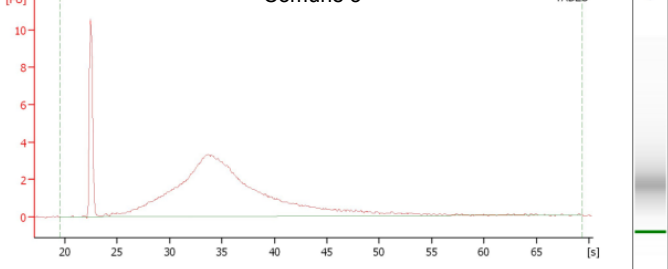

Monreal 3

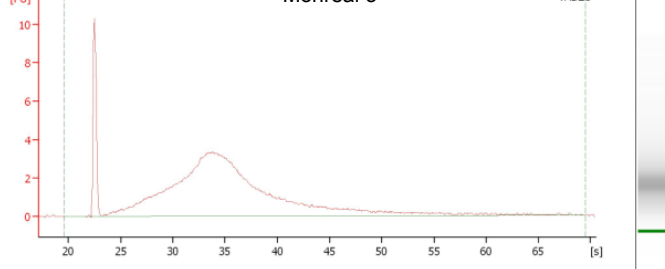

Supplement: Additional file 1 — Agilent 2100 Bioanalyzer profiles of amplified RNAs (aRNAs). Profiles show the size distribution of aRNAs produced from the different biological replicates after two rounds of amplification. Top central, RNA size marker ladder (RNA 6000 Ladder, Applied Biosystems). On the right of each electropherogram is a gel image generated from aRNAs with a smear ranging from 200 to 2000 bp and a peak around 600 bp. [file 1471-2229-12-20-S1.PDF]

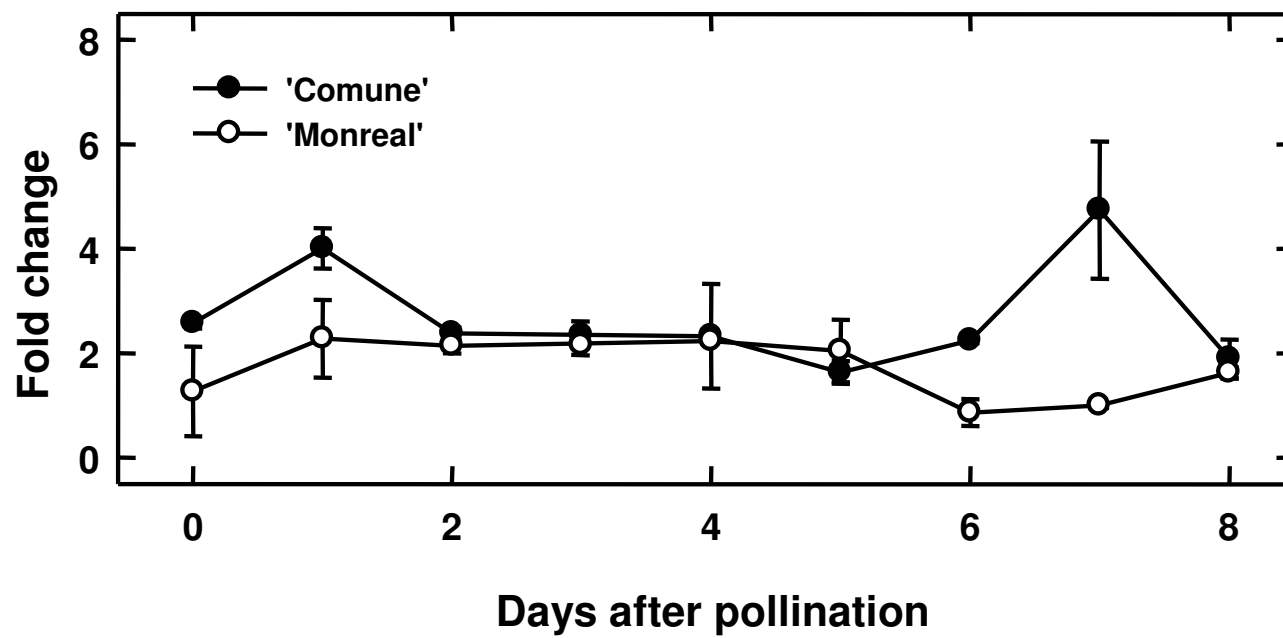

Supplement: Additional file 2 — Real time expression patterns of DELLA gene during pollen-pistil interaction in the self-compatible ('Monreal') and the self-incompatible ('Comune') genotypes. [file 1471-2229-12-20-S2.PDF]
