# Supplementary material for: Comparative transcriptome analysis of stylar canal cells identifies novel candidate genes implicated in the self-incompatibility response of Citrus clementina
Source: BMC Plant Biol. 2012 Feb 14;12:20. doi: 10.1186/1471-2229-12-20 (PMC3305554; doi:10.1186/1471-2229-12-20)
Supplement: Additional file 3 — List of primer pairs used for the real time quantitative RT-PCR analysis and for sequencing. [file 1471-2229-12-20-S3.PDF]

Primer pairs used for qRT-PCR analysis

UNIGENE ID	PRIMER FORWARD	PRIMER REVERSE
Cit.7568.1.S1_at	CGAGGTGTTGTTCTAGTGAAGC	CATTCCCACACACGCTTTC
Cit.11563.1.S1_at	CCGTAATGAACCATTTGAAGG	TAAGCAGCCGGAGCATAATC
Cit.5456.1.S1_at	AATAGTGGGACATGCACCTTATG	ATGATTGGTACATTAAGTCACTCTTGG
Cit.5776.1.S1_s_at	CTCCGGCTGCATGAATAAAC	TCATCATCTTGCCCACTCAC
Cit.7855.1.S1_at	CGTCTTATGCCCTCCTGTG	TTGACCCATTAGCGTTAGCAC
Cit.4399.1.S1_s_at	AATCACTTCATCCCGACAGC	AGGACCAAACAACCGTCTTG
Cit.24884.1.S1_at	GAGGTCAACCACAACACGAAC	GACATTCAGCTCCAGCCTTC
Cit.7174.1.S1_at	GCGTGTGCTTGTTTCAGC	ACTGGAGGCAATGGATGAC
Cit.19302.1.S1_at	CGGTGTGTTATTCGGATGTG	GGCTAACCAAGCCAATGAAG
Cit.18732.1.S1_s_at	AGGAACAGCTTCCACGTTTC	CTTCTTTGTTGCCGCATTG
Cit.12037.1.S1_at	TAGCCACAGGGTACATTTGC	CAGCGACGAGGAGGAGTATG
Cit.18491.1.S1_at	AGTACCTGGAGGGAAAATGTTT	CCGCTTTGACAATTTTATTCC
Cit.7192.1.S1_at	TTAGGTGCGGGTATCATGG	CTTAAACCGAGCCCAATGTC
Cit.1968.1.S1_s_at	AATTTCAGACGCATGGAAGC	GCGTTGGTTGTCATCGTAAC
Cit.9890.1.S1_s_at	GAAAGGTGCAAGCAAGCAG	AAGTCCCAGAAGGCACACAC
Cit.29299.1.S1_at	AGGAAGGACGCTTTCAGAGG	TCCAAAGAGCGCAGACTAGC
Cit.8702.1.S1_s_at	TGATCCGCCTCCAACAATAG	TGAGAAGAGTTGCCCTTACG
DELLA	CCACGGCTTCACATCCTC	AGAAATCGGGTTGCGACTC
AFFX-Cit-ubq11-3_x_at	GGATGGCCGTACTTTAGCTG	AGCAGAGCAGAACAGACACG

Primer pairs for sequencing the region surrounding the cit.7568 locus

UNIGENE ID	PRIMER FORWARD	PRIMER REVERSE
Amplicon 1	TGCAGCCAAATTTGAGTTTG	GTGATTCCCACCTCCTCTTG
Amplicon 2	TGTCCTCCGATTACCTCTGG	GAAAATGTTGCAAGTACCTGAGTC
Amplicon 3	AGTTAGGGTTGCGAAAGCG	CGAAGGAAATTGGATTGACG
